# MicroRNA-27b alleviates septic cardiomyopathy by targeting the Mff/MAVS axis

**DOI:** 10.3389/fcimb.2025.1588461

**Published:** 2025-07-22

**Authors:** Xincai Wang, Long Huang, Jingqing Xu, Min Li, Hongxuan Zhang, Yuqing Yao, Yaqing Liu, Xingsheng Lin, Xiuling Shang

**Affiliations:** ^1^ Department of Critical Care Medicine, Shengli Clinical Medical College of Fujian Medical University, Fuzhou University Affiliated Provincial Hospital, Fujian Provincial Hospital, Fujian Provincial Center for Critical Care Medicine, Fujian Provincial Key Laboratory of Critical Care Medicine, Fuzhou, China; ^2^ Shengli Clinical Medical College of Fujian Medical University, Fuzhou, China

**Keywords:** inflammation, microRNA-27b, mitochondrial antiviral signaling protein, mitochondrial fission factor, septic cardiomyopathy

## Abstract

**Objective:**

To investigate the protective role of microRNA-27b (miR-27b) in septic cardiomyopathy (SCM) and its regulatory mechanism on the mitochondrial fission factor (Mff)/mitochondrial antiviral signaling protein (MAVS) axis.

**Methods:**

Transcriptome data from septic patients’ cardiac tissues (GSE79962) were analyzed. Serum miR-27b expression was measured in SCM patients (n=11), sepsis-only patients (n=22), and healthy controls (n=30). Mouse SCM model and HL-1 cardiomyocyte model were established by lipopolysaccharide (LPS) induction. The molecular mechanism was investigated using miR-27b agonist/antagonist and Mff intervention, combined with RT-qPCR, Western blot, immunofluorescence, and transmission electron microscopy.

**Results:**

Bioinformatics analysis revealed significant downregulation of miR-27b in SCM cardiac tissues (log2FC=-3.9, *P*<0.001). Clinical validation showed lower miR-27b expression in SCM patients’ serum compared to sepsis-only patients and healthy controls (*P*<0.05). LPS-induced SCM model exhibited cardiac dysfunction, myocardial injury, mitochondrial abnormalities, decreased miR-27b expression, and increased Mff and MAVS levels. miR-27b targeted Mff to maintain mitochondrial homeostasis, thus attenuating LPS-induced cardiomyocyte inflammation and apoptosis, while Mff overexpression reversed this protective effect.

**Conclusion:**

miR-27b alleviates myocardial injury and inflammation in SCM by targeting the Mff/MAVS axis to maintain mitochondrial homeostasis, representing a potential novel therapeutic target for SCM.

## 1 Introduction

Sepsis is a systemic inflammatory response triggered by infection, characterized by dysregulated host immune responses leading to multiple organ dysfunction (Singer et al., 2016) Despite advances in medical technology, sepsis remains a significant global health burden with approximately 11 million deaths annually (Rudd et al., 2020) Among various complications, sepsis-induced cardiac dysfunction is a primary contributor to mortality in septic patients (L’Heureux et al., 2020; Hollenberg and Singer, 2021). Mitochondrial dynamics imbalance plays a central role in the pathogenesis of septic cardiomyopathy (SCM). Our previous research demonstrated that SRV2 protein promotes mitochondrial fission in sepsis by activating the Mst1-Drp1 signaling pathway (Shang et al., 2020). In LPS-induced SCM models, Mst1 upregulation activates the Drp1/F-actin pathway, resulting in excessive mitochondrial fission, metabolic dysfunction, and cellular apoptosis (Shang et al., 2019). Mitochondria not only serve as energy metabolism centers but also play crucial roles in infectious and innate immune responses (Rambold and Pearce, 2018; Shang et al., 2020; Mokhtari et al., 2023).

Mitochondrial antiviral signaling protein (MAVS) is a key immune regulatory factor localized on the outer mitochondrial membrane that induces inflammatory cascade reactions when activated (Kim et al., 2021). Recent studies indicate that MAVS can also sense non-viral pathogens, cellular damage, and metabolic stress (Trishna et al., 2023). Mitochondrial fission, fusion, and autophagy processes participate in regulating MAVS signal transduction (Hanada et al., 2024), while MAVS activity is modulated by mitochondrial energy status, establishing a molecular link between energy metabolism and immune responses (Lin et al., 2020; Ailenberg et al., 2022). However, the specific mechanism of MAVS-mediated mitochondrial dynamics changes and innate immune injury in SCM remains unclear.

To investigate the molecular mechanisms of mitochondrial dysfunction in SCM, we analyzed transcriptome data from septic patients’ cardiac tissues (GSE79962). The analysis identified 2,677 differentially expressed genes (adjusted *P*<0.05), with miR-27b showing significant downregulation. As a non-coding RNA, miR-27b plays an important role in regulating mitochondrial dynamics: in hepatocytes, miR-27b can inhibit mitochondrial fission factor (Mff) (Tak et al., 2014), a key protein regulating mitochondrial division (Xiong et al., 2022). Inhibition of Mff overexpression reduces mitochondrial fission, maintains mitochondrial integrity, and attenuates apoptosis (Zhou et al., 2020). Additionally, miR-27 exhibits anti-inflammatory effects: in sepsis-induced liver injury, it protects through targeting TAB3 to inhibit the NF-κB signaling pathway (Yang et al., 2018) and can reduce excessive inflammatory responses caused by infection (Liang et al., 2018; Lu et al., 2020).

Based on these findings, we hypothesized that miR-27b might participate in SCM pathogenesis by regulating the Mff/MAVS axis. This study aimed to elucidate the role of the miR-27b/Mff/MAVS signaling axis in SCM through clinical sample analysis, animal model verification, and molecular mechanism exploration, potentially providing new theoretical basis for SCM diagnosis and treatment.

## 2 Results

### 2.1 Transcriptome analysis reveals significant downregulation of miR-27b in septic cardiac tissues

**Figure 1:**
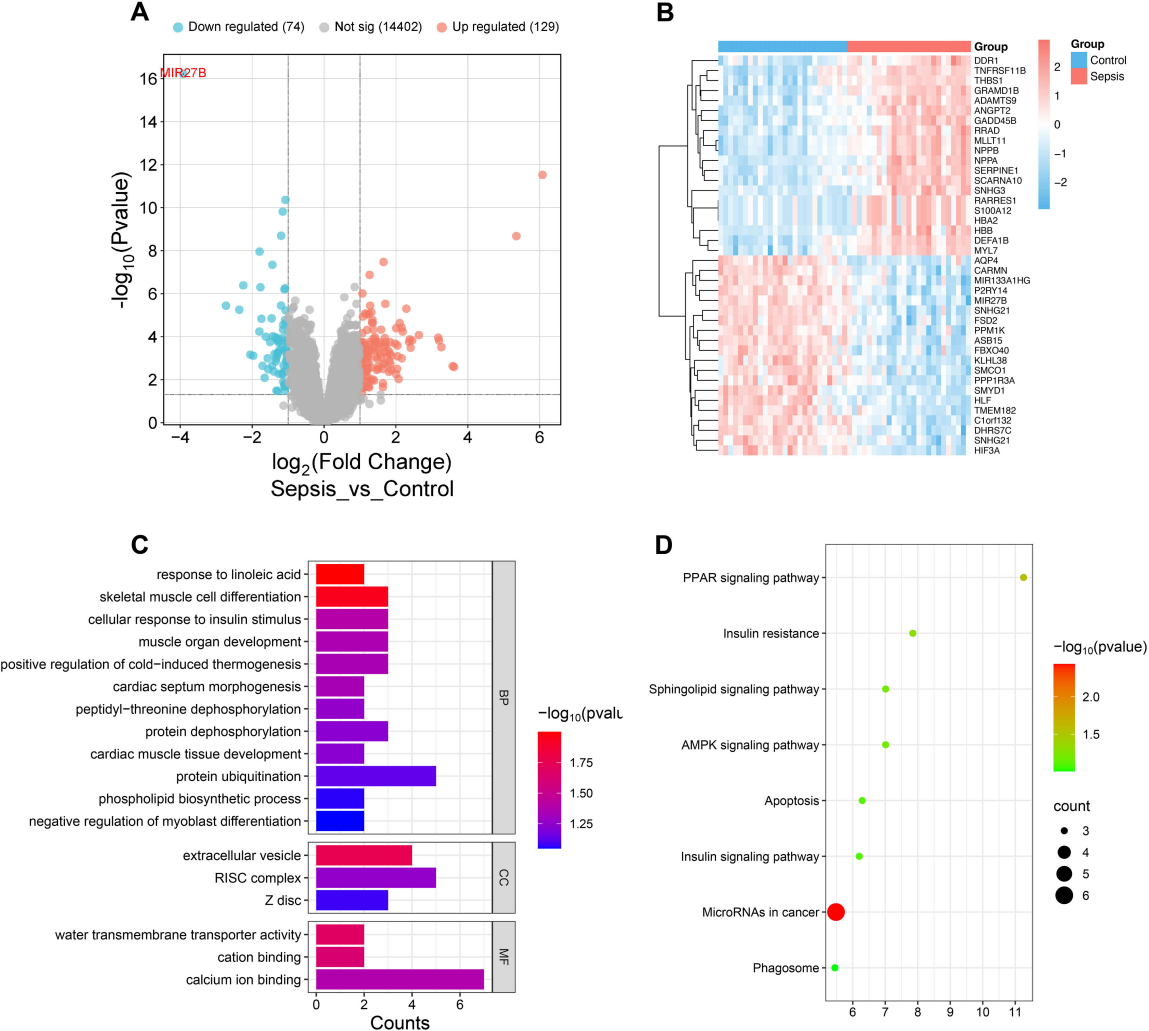
Transcriptome analysis reveals miR-27b expression in septic cardiac tissues. **(A)**, Volcano plot showing distribution of differentially expressed genes in cardiac tissues from sepsis patients. **(B)**, Heatmap showing top 20 up/down-regulated genes in control and sepsis groups. **(C)**, GO enrichment analysis showing enrichment of differentially expressed genes in biological processes (BP), cellular components (CC), and molecular functions (MF). **(D)**, KEGG pathway enrichment analysis.

To explore key regulatory molecules associated with SCM, we analyzed transcriptome data from septic patients’ and healthy controls’ cardiac tissues in the GEO database (GSE79962). Differential expression analysis identified 2,677 differentially expressed genes (adjusted P<0.05), including 74 downregulated and 126 upregulated genes (|log2FoldChange|>1, adjusted P<0.05) (**Figure 1A**). Notably, miR-27b exhibited the most significant downregulation in the sepsis group (log2FoldChange=-3.9, adjusted P<0.001) (**Figure 1A**). Heatmap showing top 20 up/down-regulated genes in control and sepsis groups revealed distinct gene expression patterns between the two groups (**Figure 1B**). GO and KEGG enrichment analyses revealed that these differentially expressed genes were enriched in multiple biological processes and signaling pathways related to mitochondrial function, energy metabolism, and cell apoptosis (**Figures 1C, D**). These results suggest that miR-27b downregulation may influence SCM pathogenesis by affecting mitochondrial energy metabolism and signal transduction processes.

### 2.2 miR-27b is significantly downregulated in SCM patients’ serum

**Figure 2:**
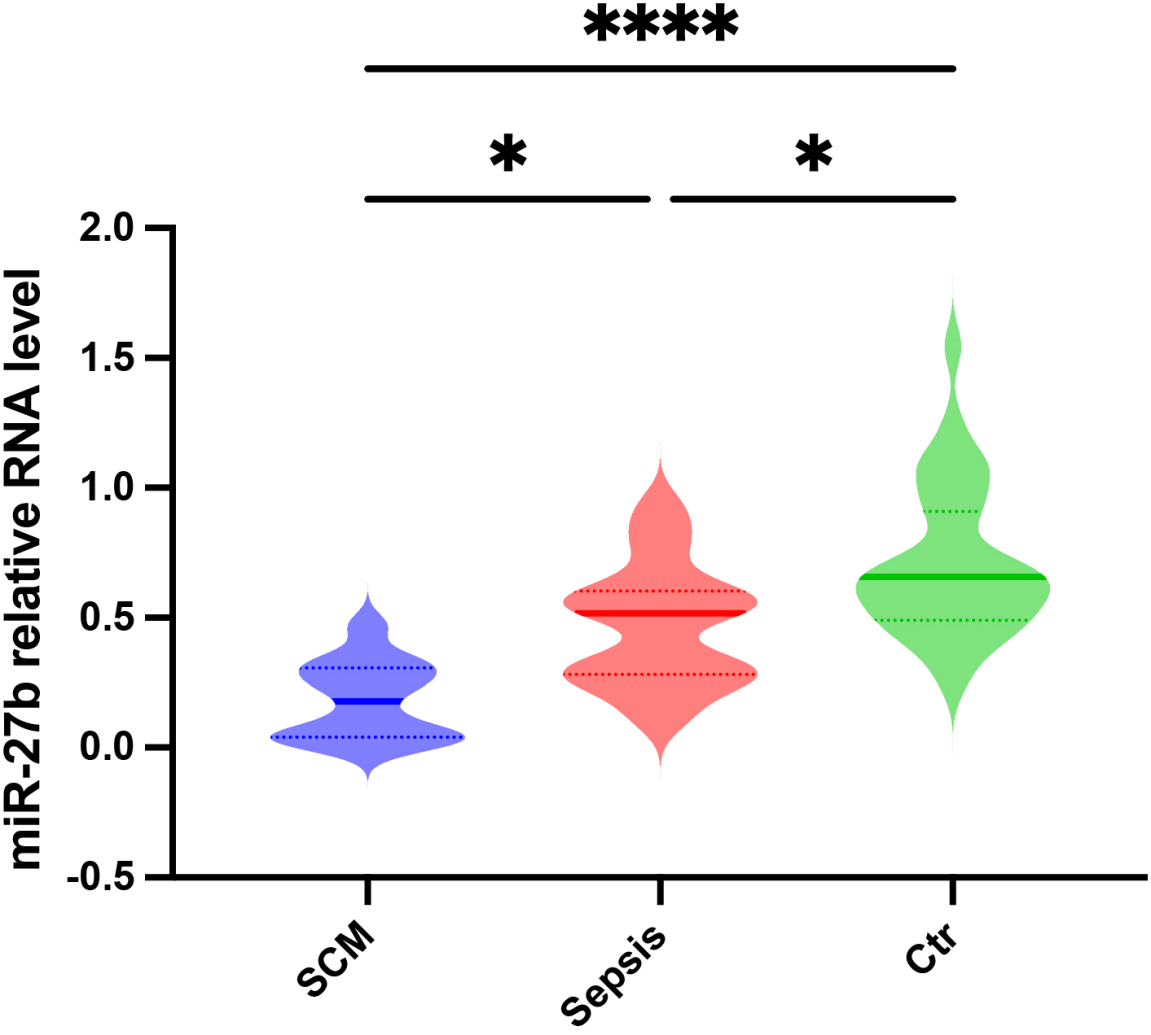
Relative miR-27b expression levels in serum from SCM patients, sepsis patients, and healthy controls. Error bars represent mean ± s.d. *P<0.05, ****P<0.0001, two-tailed Student’s t-test.

To validate the bioinformatic analysis results and assess miR-27b expression patterns in clinical samples, we collected serum from SCM patients, sepsis patients without cardiac dysfunction, and healthy controls (Ctr). The detailed patient selection process is illustrated in **Supplementary Figure S1**. The clinical characteristics of the SCM and sepsis group are summarized in **Supplementary Table S1**. RT-qPCR analysis revealed that serum miR-27b expression levels were significantly reduced in sepsis patients compared to healthy controls (*P*<0.05). More importantly, SCM patients exhibited a further reduction in serum miR-27b levels, which were significantly lower than both the healthy control group (*P*<0.0001) and the sepsis-only group (*P*<0.05) (**Figure 2**). These findings indicate that miR-27b expression levels correlate with disease severity, suggesting its potential involvement in SCM pathogenesis and diagnostic value.

### 2.3 Phenotypic validation of LPS-induced myocardial injury and inflammatory response in mice

**Figure 3:**
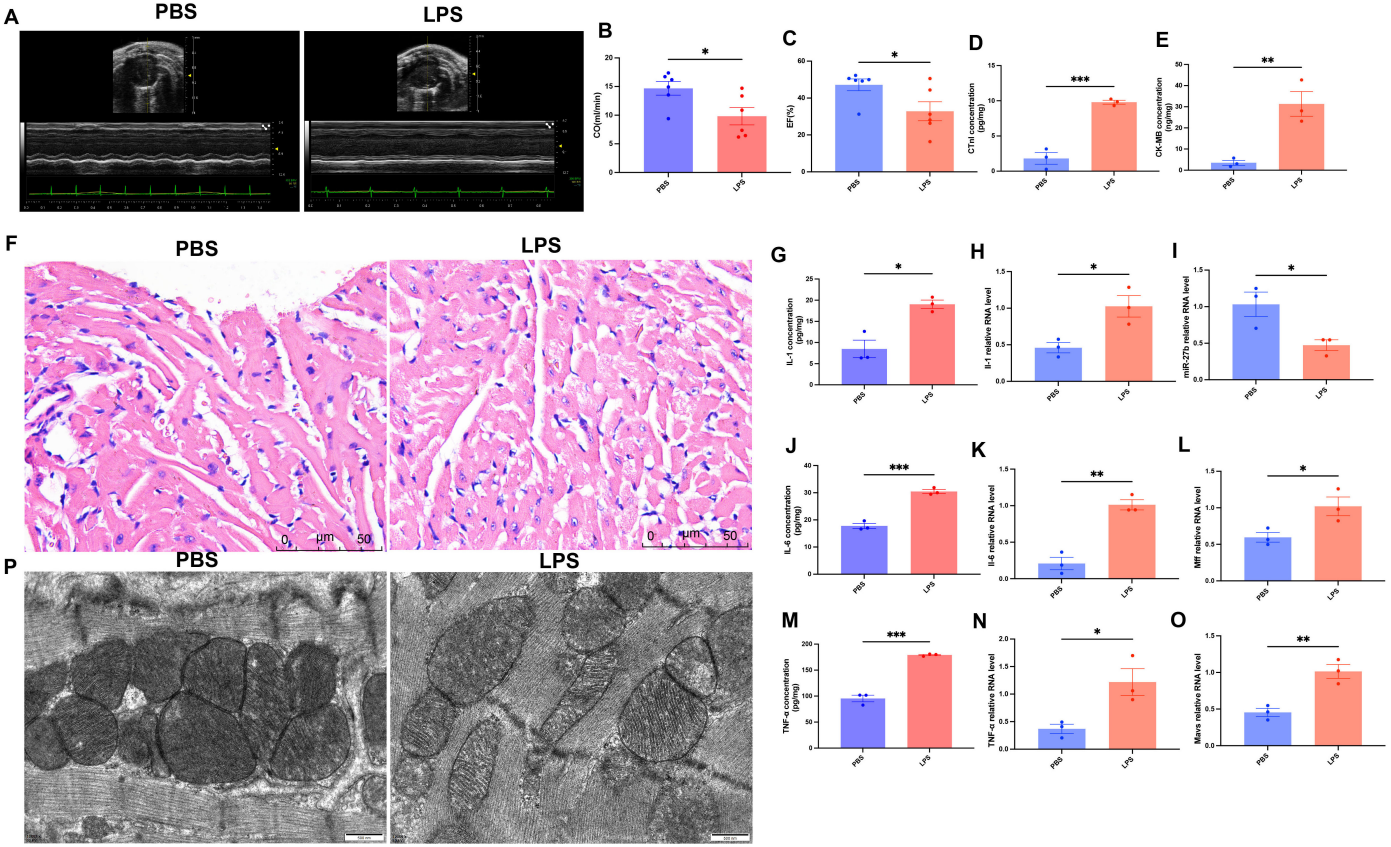
Phenotypic validation of LPS-induced myocardial injury and inflammatory response in mice. **(A)**, Representative echocardiographic images. **(B, C)**, Quantitative analysis of cardiac output (CO) and ejection fraction (EF) (n=6/group). **(D, E)**, ELISA detection of cardiac tissue cTnI and CK-MB levels (n=3/group). **(F)**, H&E staining of cardiac tissues, scale bar=50 μm. **(G, J, M)**, ELISA detection of cardiac tissue inflammatory factors (IL-1β, IL-6, TNF-α) (n=3/group). **(H, K, N)**, RT-qPCR detection of inflammatory factor mRNA expression levels in cardiac tissues (n=3/group). **(I, L, O)**, RT-qPCR detection of miR-27b, Mff, and MAVS expression levels in cardiac tissues (n=3/group). **(P)**, Transmission electron microscopy of cardiomyocyte mitochondrial ultrastructure, scale bar=500 nm. Data are presented as mean ± s.d. *P<0.05, **P<0.01, ***P<0.001, two-tailed Student’s t-test.

To validate the LPS-induced SCM model, we performed multidimensional phenotypic analysis in mice. First, cardiac function was assessed by echocardiography (**Figure 3A**). Results showed that compared to the PBS control group, LPS-treated mice exhibited significantly reduced cardiac output (CO) (14.8 ± 1.2 vs 9.2 ± 1.5 ml/min, *P*<0.05) (**Figure 3B**) and ejection fraction (EF) (45.6 ± 3.2 vs 30.5 ± 4.1%, *P*<0.05) (**Figure 3C**). Serological tests revealed significantly elevated levels of myocardial injury markers, cardiac troponin I (cTnI) and creatine kinase-MB (CK-MB), in the LPS group (**Figures 3D, E**, *P*<0.001 and *P*<0.01, respectively).

Morphological comparison of heart tissues using H&E staining showed that PBS-treated hearts maintained intact structure with regularly arranged myocardial fibers, normal nuclear morphology with uniform staining, and minimal interstitial edema or inflammatory cell infiltration. In contrast, LPS-treated hearts exhibited significant pathological changes: disorganized myocardial fibers with wavy deformation in some areas, swollen cardiomyocytes with granular cytoplasmic changes suggesting mitochondrial structural abnormalities, widened intercellular spaces indicating tissue edema, and notable inflammatory cell infiltration (**Figure 3F**). Additionally, ELISA results showed significantly elevated cardiac tissue levels of IL-1β, IL-6, and TNF-α in LPS-treated mice (**Figures 3G, J, M**, all *P*<0.05). RT-qPCR results confirmed that mRNA expression levels of these inflammatory factors were also significantly upregulated in cardiac tissues (**Figures 3H, K, N**, all *P*<0.05).

Transmission electron microscopy revealed that control mice had cardiomyocyte mitochondria with typical oval or rod-like shapes and clear, intact cristae, whereas LPS-treated mice exhibited mitochondrial swelling with disorganized and partially fragmented cristae (**Figure 3P**). Further RT-qPCR detection showed that compared to the control group, miR-27b expression was significantly decreased in LPS-treated cardiac tissues (**Figure 3I**, *P*<0.05), while its target gene Mff (**Figure 3L**, *P*<0.05) and downstream molecule MAVS (**Figure 3O**, *P*<0.01) were significantly upregulated.

These results indicate successful establishment of the SCM model, characterized by significant cardiac dysfunction, myocardial injury, inflammatory response, and mitochondrial abnormalities. Concurrently, we observed significant alterations in the miR-27b/Mff/MAVS signaling axis, suggesting its potential involvement in disease development.

### 2.4 miR-27b reduces mitochondrial fission by targeting Mff

**Figure 4:**
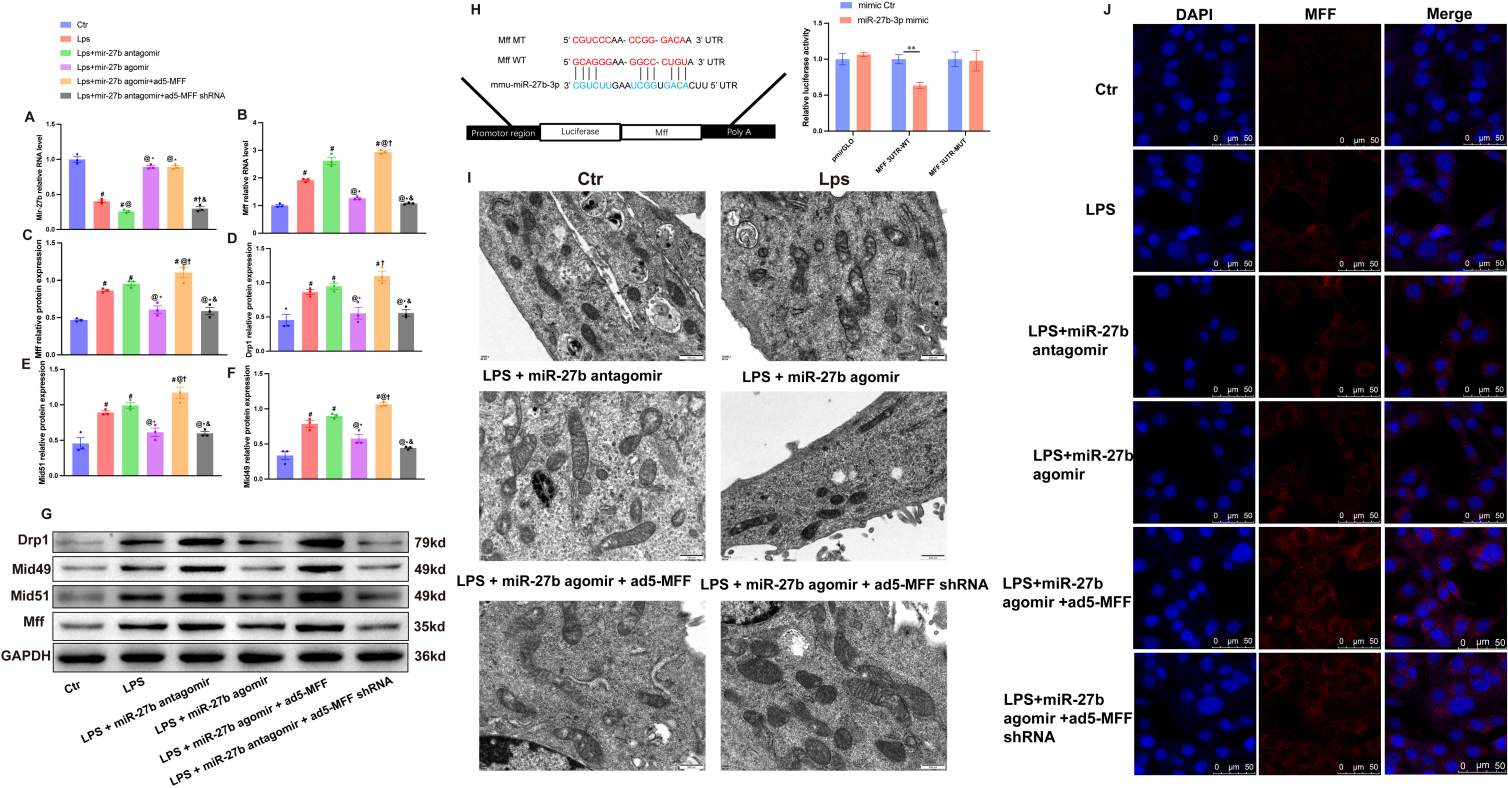
miR-27b reduces mitochondrial fission by targeting Mff. **(A, B)**, RT-qPCR detection of miR-27b and Mff mRNA expression levels in each cell group. **(C–F)**, Quantitative analysis of Mff, Drp1, Mid49, and Mid51 protein expression levels by Western blot. **(G)**, Representative Western blot bands for each group. **(H)**, Dual-luciferase reporter assay verifying the targeting relationship between miR-27b and Mff; upper panel shows wild-type (WT) and mutant-type (MT) Mff 3’UTR sequence diagrams. **(I)**, Transmission electron microscopy observation of mitochondrial ultrastructure in each cell group, scale bar=500 nm. **(J)**, Confocal laser scanning microscopy observation of MFF expression and subcellular localization in each cell group, scale bar=50 μm. #*P*<0.05 vs Ctr group; @*P*<0.05 vs LPS group; *P<0.05 vs LPS+miR-27b antagomir group; †*P*<0.05 vs LPS+miR-27b agomir group; &*P*<0.05 vs LPS+miR-27b agomir+ad5-MFF group; **P<0.01. One-way ANOVA followed by Tukey’s *post hoc* test.

To investigate the regulatory mechanism of miR-27b on cardiomyocyte mitochondrial fission, we performed a series of intervention experiments on HL-1 cardiomyocytes. RT-qPCR analysis showed that LPS treatment significantly decreased miR-27b expression while increasing Mff mRNA levels in cardiomyocytes (Figures 4A, B). Results showed that compared to the control group, LPS treatment significantly affected mitochondrial fission-related protein expression. Western blot analysis revealed significant increases in protein expression levels of mitochondrial fission factors Mff, Drp1, Mid49, and Mid51 (**Figures 4C–G**).

Further intervention experiments demonstrated that miR-27b agonist (agomir) treatment significantly inhibited LPS-induced upregulation of mitochondrial fission-related genes, while the miR-27b inhibitor (antagomir) further exacerbated the LPS-induced effects. To verify Mff’s role in this process, we downregulated Mff expression via adenovirus-mediated shRNA in cells treated with LPS+miR-27b inhibitor. Results showed that Mff knockdown significantly reversed the increased expression of mitochondrial fission-related proteins caused by miR-27b inhibition (**Figures 4C–G**).

Transmission electron microscopy showed normal mitochondrial morphology in control cardiomyocytes, while LPS-treated cells exhibited numerous fragmented mitochondria. miR-27b agonist significantly improved mitochondrial morphology, whereas inhibitor exacerbated mitochondrial fragmentation. Mff knockdown partially reversed the mitochondrial morphological abnormalities caused by miR-27b inhibition (**Figure 4I**).

To further verify the direct regulatory relationship between miR-27b and Mff, we performed dual-luciferase reporter assays. Results showed that compared to the control group, miR-27b significantly inhibited luciferase activity of wild-type Mff 3’UTR but had no significant effect on the mutant type (**Figure 4H**). Immunofluorescence staining also showed that LPS treatment increased Mff expression, miR-27b agonist inhibited this increase, and inhibitor further enhanced Mff expression. In the presence of miR-27b inhibitor, Mff knockdown significantly reduced its expression level (**Figure 4J**).

These results demonstrate that miR-27b directly targets Mff to regulate mitochondrial fission. Downregulation of miR-27b leads to increased Mff expression, promoting excessive mitochondrial fission, which may represent an important mechanism in SCM pathogenesis.

### 2.5 Mff overexpression reverses the protective effects of miR-27b against inflammation and apoptosis

**Figure 5:**
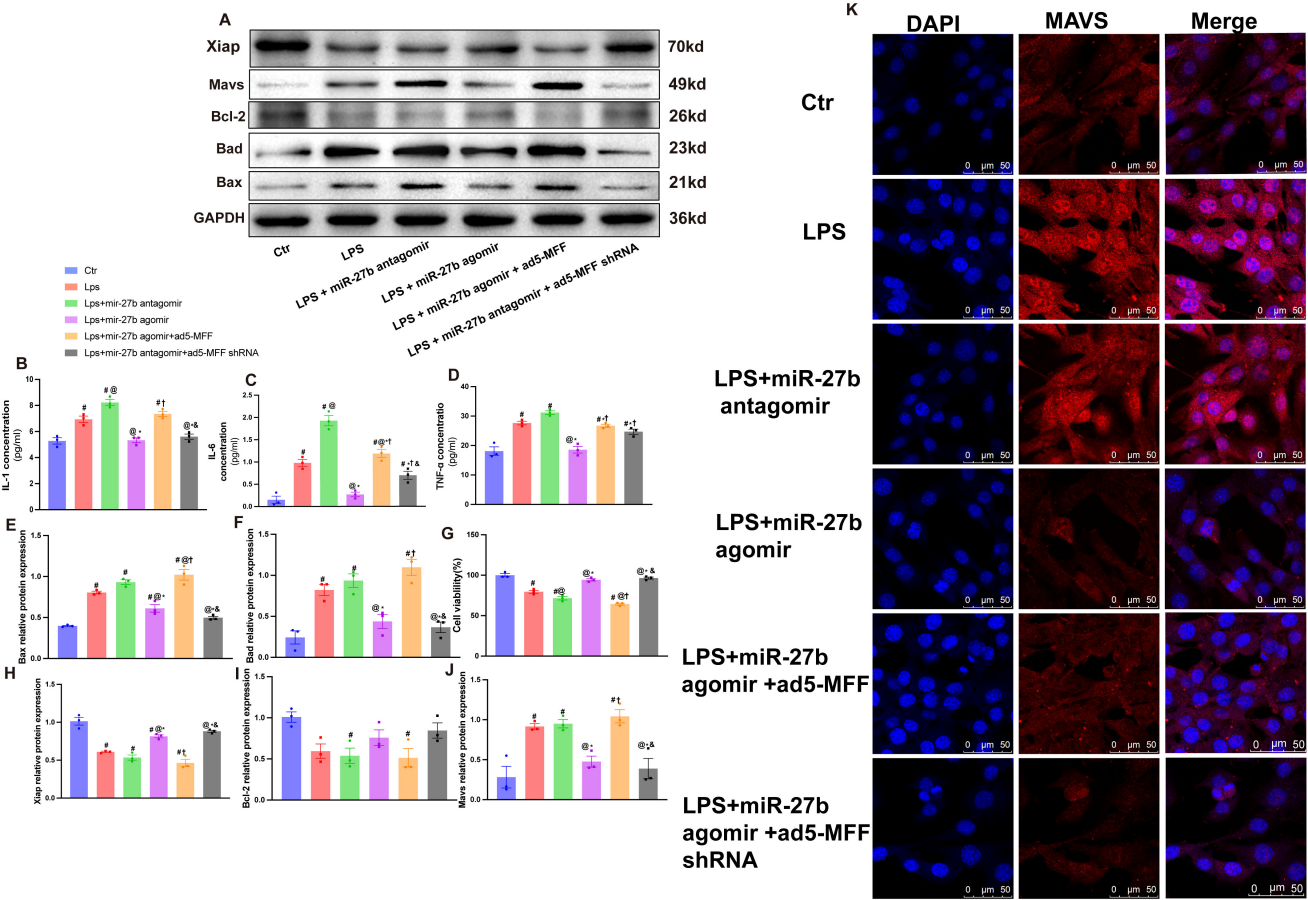
Mff overexpression reverses the protective effects of miR-27b against inflammation and apoptosis. **(B–D)**, ELISA detection of IL-1β, IL-6, and TNF-α levels in each cell group. **(A, E, F)**, Western blot quantitative analysis of Bad and Bax protein expression. **(G)**, CCK-8 detection of cell viability in each group. **(A, H, I)**, Quantitative analysis of Bcl-2 and XIAP protein expression. **(J)**, Quantitative analysis of MAVS protein expression. **(K)**, Immunofluorescence staining detecting MAVS expression and localization, scale bar=50 μm. Data are presented as mean ± s.d. (n=3 independent experiments). #*P*<0.05 vs Ctr group; @*P*<0.05 vs LPS group; *P<0.05 vs LPS+miR-27b antagomir group; †*P*<0.05 vs LPS+miR-27b agomir group; &*P*<0.05 vs LPS+miR-27b agomir+ad5-MFF group; **P<0.01. One-way ANOVA followed by Tukey’s *post hoc* test.

To explore the mechanism of the miR-27b/Mff/MAVS signaling axis in SCM, we further examined changes in inflammatory factors and apoptosis-related molecules. ELISA results showed that compared to the control group, LPS treatment significantly upregulated IL-1β, IL-6, and TNF-α levels (**Figures 5B–D**). Notably, miR-27b agonist significantly inhibited LPS-induced inflammatory factor expression, while miR-27b inhibitor further enhanced the inflammatory response. Importantly, Mff overexpression in LPS+miR-27b agonist-treated cells reversed the anti-inflammatory effects of miR-27b.

Regarding apoptosis, we examined the expression levels of pro-apoptotic proteins Bad and Bax (**Figures 5E, F**) and anti-apoptotic proteins Bcl-2 and XIAP (**Figures 5H, I**). Results showed that LPS significantly increased pro-apoptotic protein levels, decreased anti-apoptotic protein expression, and reduced cell viability (**Figure 5G**). miR-27b agonist partially reversed these changes, while inhibitor exacerbated the apoptotic trend.

Immunofluorescence staining revealed that compared to the control group, LPS treatment significantly increased MAVS expression and altered its cellular localization. miR-27b agonist inhibited MAVS upregulation, while inhibitor further enhanced MAVS expression. Notably, in the presence of LPS+miR-27b agonist, Mff overexpression reversed miR-27b’s inhibitory effect on MAVS expression, while Mff knockdown attenuated this effect (**Figure 5K**). MAVS protein levels were further validated by Western blot (**Figure 5J**).

These results indicate that miR-27b modulates inflammatory responses and apoptotic processes by regulating the Mff-MAVS axis. Mff overexpression can reverse the protective effects of miR-27b, revealing the important role of the miR-27b/Mff/MAVS signaling axis in SCM pathogenesis and providing potential new therapeutic targets.

## 3 Discussion

SCM is a life-threatening complication of sepsis that significantly increases mortality risk (Hollenberg and Singer, 2021; Carbone et al., 2022). Through bioinformatic analysis, clinical samples, animal models, and molecular mechanism studies, this research systematically elucidates the regulatory role of the miR-27b/Mff/MAVS signaling axis in SCM pathogenesis. Bioinformatic analysis suggests that miR-27b downregulation may affect mitochondrial energy metabolism and signal transduction processes in SCM. In our clinical cohort, we discovered for the first time that SCM patients exhibit more significant miR-27b downregulation compared to sepsis-only patients. Animal experiments and cell models further validated the biological significance of this finding: miR-27b downregulation was accompanied by significant myocardial injury and inflammatory responses, while miR-27b overexpression effectively alleviated LPS-induced cardiomyocyte dysfunction.

miR-27b expression levels are closely associated with disease progression. Previous studies have shown that in viral infection models, miR-27b-3p downregulation weakened host antiviral immune responses, while restoring its expression through miR-27b mimics significantly enhanced host defense capabilities and reduced mortality (Wang et al., 2022). In HCV infection research, miR-27b inhibited viral replication and attenuated inflammatory injury by regulating aquaporin AQP11 (Sakurai et al., 2019). More importantly, miR-27b possesses multiple protective effects: inhibiting TNF-α-induced mitochondrial oxidative stress and cellular apoptosis (D’Onofrio et al., 2023), promoting angiogenesis, reducing fibrosis, and regulating inflammatory responses (Veliceasa et al., 2015). These evidences collectively support the protective role of miR-27b in SCM.

Molecular mechanism studies revealed that Mff is a direct target of miR-27b, and its overexpression can reverse the cardioprotective effects mediated by miR-27b. In ischemia-reperfusion injury, Mff interacts with VDAC1 to regulate mitochondrial permeability transition pore opening, promoting mitochondrial fission and cellular apoptosis (Wang et al., 2024). SIRT1 upregulation improves cardiac function by inhibiting Mff-mediated mitochondrial fission (Xu et al., 2024), while in metabolic cardiomyopathy, Mff activation exacerbates mitochondrial dysfunction (Bekhite et al., 2021). Our study is among the first to elucidate the molecular mechanism of Mff-induced mitochondrial dysfunction in SCM.

As a mitochondrial outer membrane protein (Manes and Nita-Lazar, 2021), MAVS was confirmed to be regulated by Mff in this study. *In vitro* research has demonstrated that Mff can promote the formation of active MAVS clusters on mitochondria (Hanada et al., 2020). MAVS regulates cellular apoptosis through interaction with AMBRA1 (Lin et al., 2022), activates the NF-κB pathway during viral infections to trigger inflammatory factor release (Pinto et al., 2014), and inhibiting MAVS can significantly reduce inflammatory responses and improve sepsis outcomes (Hsu et al., 2023). These findings reveal the critical role of MAVS in connecting mitochondrial dysfunction with inflammatory immune responses.

In addition to miR-27b, several other microRNAs have been identified as potential therapeutic targets in septic cardiomyopathy (SCM). miR-21 promotes inflammation and mitochondrial dysfunction via SORBS2 and NLRP3 activation (Wang et al., 2016b), while miR-146a attenuates cardiac injury by suppressing TLR4/NF-κB signaling (Xie et al., 2019). miR-155 improves cardiac function in CLP-induced models by modulating immune pathways such as JNK and β-arrestin 2 (Zhou et al., 2017). Compared to the miR-27b/Mff/MAVS axis, which mainly regulates mitochondrial dynamics and inflammation, these microRNAs act through distinct mechanisms. Their complementary roles highlight the need for broader investigation into miRNA-based interventions in SCM.

Interestingly, ferroptosis has been found to play an important role in the pathogenesis of septic cardiomyopathy (Kong et al., 2022; Fang et al., 2023; Zeng et al., 2023). Although there is no direct evidence showing that miR-27b regulates ferroptosis in SCM, studies by Xun Lu et al. demonstrated that miR-27a (belonging to the same family as miR-27b) can modulate ferroptosis via targeting SLC7A11 in NSCLC cells, indicating the significant role of miR-27a-3p in ferroptosis. Considering the high homology between miR-27b and miR-27a (Kim et al., 2016), this suggests that the miR-27b/Mff/MAVS signaling axis may be related to ferroptosis in addition to regulating inflammatory responses and apoptosis.

Ferroptosis is closely related to reactive oxygen species (ROS), and mitochondria are important sites for ROS production. Although our study did not directly measure ROS levels, research by Nunzia D’Onofrio demonstrates that miR-27b has been shown to attenuate mitochondrial oxidative stress and inflammation in endothelial cells (D’Onofrio et al., 2023). Future research could further explore the specific mechanisms of miR-27b in ROS generation and regulation in SCM.

However, this study has several limitations. In clinical research: (1) The small sample size of SCM patients (n=11) may reduce the robustness of statistical analysis and generalizability of conclusions. And the data in this study came from only a single medical center, which may limit the generalizability of the results, especially considering that treatment regimens and patient characteristics may vary across different medical centers. To address this, we plan to conduct future studies based on larger, multi-center clinical cohorts that can better capture the heterogeneity of septic cardiomyopathy (SCM) and validate the diagnostic and prognostic potential of miR-27b; (2) Due to critical illness severity, cardiac tissue biopsy specimens could not be obtained for direct verification, relying instead on serum sample analysis which may not fully reflect molecular changes in cardiac tissue; (3) Prospective cohort studies evaluating the relationship between miR-27b expression levels and short- and long-term prognosis are lacking; (4) Non-infectious myocardial injury control groups, such as heart failure, were not included, limiting the assessment of miR-27b as an SCM-specific marker.

In experimental research: (1)Our study utilized a lipopolysaccharide (LPS)-induced septic cardiomyopathy (SCM) mouse model, which is widely applied to simulate early sepsis-related inflammatory responses and cardiac dysfunction. Through intraperitoneal injection of LPS, we could induce systemic inflammatory responses, myocardial depression, and mitochondrial dysfunction similar to early human SCM. This model offers advantages of high reproducibility, low cost, and controllable disease progression, making it particularly suitable for investigating miRNA-mediated mitochondrial dynamics and innate immune signaling pathway mechanisms. However, we acknowledge that this model cannot fully replicate the complex pathological processes of human sepsis, particularly lacking differences in immune responses caused by various pathogens like bacteria and fungi. Notably, Wang et al. employed the cecal ligation and puncture (CLP) model, which represents a polymicrobial sepsis model (Sun et al., 2021). Their study demonstrated that exosomes derived from mesenchymal stem cells (MSCs) overexpressing miR-27b significantly attenuated the inflammatory response in the myocardium of CLP-induced septic mice. Mechanistically, miR-27b exerted this protective effect by targeting JMJD3 and inhibiting the NF-κB/p65 signaling pathway, thereby reducing the expression of inflammatory cytokines. These findings suggest that miR-27b plays a protective role across different experimental models of sepsis; (2) This study only performed H&E staining analysis on PBS and LPS mouse groups to verify model establishment, therefore the *in vivo* experiments to some extent limited the morphological validation of miR-27b regulatory effects. (3)This study primarily focused on miR-27b’s regulation of Mff but did not exclude the influence of other potential targets through genome-wide methods; (4) Although a functional association between Mff and MAVS was observed, the direct molecular interaction mechanism requires further elucidation; (5) Systematic evaluation of dynamic expression changes of miR-27b at different sepsis stages and its temporal relationship with disease progression was not performed.

In translational research: (1) Systematic evaluation of miR-27b’s safety, efficacy, and administration routes as a therapeutic target is lacking; (2) Specific drug intervention strategies targeting the Mff-MAVS axis have not been established; (3) The synergistic effects of miR-27b with existing sepsis treatment regimens and its differential effects in various pathological SCM subtypes remain unexplored; (4) Preclinical validation data in large animal models is lacking, limiting direct translational value for clinical applications.

To address these limitations, future work will involve expanding multi-center clinical cohorts, incorporating dynamic sampling to assess miR-27b changes over time, and introducing control groups such as non-infectious myocardial injury to evaluate specificity. In experimental studies, polymicrobial sepsis models and mitochondrial functional assays will be applied to validate findings across disease settings.

Importantly, the management of SCM remains largely supportive, including fluid resuscitation, vasopressor administration, and mechanical ventilation. However, these interventions primarily address hemodynamic instability and do not specifically target the underlying mitochondrial and immunometabolic dysfunctions implicated in SCM pathogenesis. Despite growing recognition of SCM as a distinct sepsis complication, there are no approved therapies that directly mitigate cardiac mitochondrial injury or inflammation. Our findings highlight miR-27b as a potential upstream modulator that regulates mitochondrial fission and innate immune signaling, suggesting that miR-27b/Mff/MAVS axis may serve as a promising molecular target to complement existing treatments.

In conclusion, this study explored the regulatory mechanism of the miR-27b/Mff/MAVS signaling axis in SCM: miR-27b maintains mitochondrial homeostasis by targeting Mff, inhibiting MAVS-mediated inflammatory cascade reactions, thereby reducing myocardial injury. This finding not only deepens our understanding of SCM pathogenesis but also provides a theoretical basis for developing new therapeutic strategies.

## 4 Methods

### 4.1 Clinical sample collection

This study enrolled sepsis patients (n=33) admitted to the ICU of Fujian Provincial Hospital and healthy controls (n=30) from September 2021 to December 2022. For critically ill patients unable to personally provide informed consent, we obtained consent from their next of kin or legal representative following Ethics Committee-approved procedures. Upon regaining consciousness, patients were re-informed and their participation was reconfirmed. This process was witnessed by research staff, documented thoroughly, and complies with the Declaration of Helsinki and institutional ethics requirements. According to the Sepsis-3 diagnostic criteria (2016), patients were divided into SCM group (n=11) and sepsis-only group (n=22). Inclusion criteria included: sepsis patients with SOFA score ≥2 and confirmed or suspected infection(1); SCM patients meeting sepsis criteria with any of the following cardiac dysfunction: LVEF<50% or GLS<-18% to -20%, E/e’>14, RFAC<35% or TAPSE<17 mm(3); All participants enrolled in this study were adults aged 18 years or older. Exclusion criteria included: prior chronic cardiac disease with NYHA class ≥III, acute coronary syndrome, myocarditis, or pericarditis, end-stage malignancy, severe trauma, pregnancy, or inability to obtain transthoracic echocardiography data. Peripheral blood (5ml) was collected, centrifuged at 3000g for 15min at 4°C to separate serum, and stored at -80°C until analysis. All clinical trial patients and volunteers signed informed consent forms, and the study was approved by the Clinical Ethics Committee of Fujian Provincial Hospital (Approval No: K2022-05-08).

### 4.2 Animal studies

Male C57BL/6J mice (6–8 weeks old, 20-22g, Beijing Sipeifu Biotechnology) were housed in SPF-grade facilities at 26 ± 2°C, 50 ± 5% humidity, with 12h light/dark cycles and free access to food and water. After one week of acclimatization, mice were randomly divided into control and SCM model groups (n=12 each). The SCM model was established by intraperitoneal injection of LPS (5 mg/kg, from E. coli O111:B4, Sigma-L4391) (Wang et al., 2016a; Li et al., 2022), while control group received equal volume of PBS. All animal procedures were approved by the Animal Ethics Committee of Fujian Provincial Hospital (Approval No: K2022-05-08).

### 4.3 Real-time quantitative PCR

Total RNA was extracted using Trizol reagent (Invitrogen, 15596026) and quantified using NanoDrop 2000. Reverse transcription was performed using PrimeScript RT Kit (TaKaRa, RR037A) according to manufacturer’s instructions. RT-qPCR was conducted using SYBR Green Master Mix (Applied Biosystems, 4309155) on ABI 7500 system. Cycling conditions: 95°C for 10min; 40 cycles of 95°C for 15s and 60°C for 1min. Relative expression was calculated using 2-ΔΔCt method (Livak and Schmittgen, 2001).

### 4.4 Western blot analysis

Total protein was extracted using RIPA buffer (containing protease and phosphatase inhibitor cocktail, Thermo Fisher, 89900), and protein concentration was determined by BCA assay (Pierce, 23225). Proteins (30μg) were separated by SDS-PAGE and transferred to 0.45μm PVDF membranes (Millipore, IPVH00010). After blocking with 5% non-fat milk (BD, 232100) for 2h at room temperature, membranes were incubated overnight at 4°C with primary antibodies: Bad (1:1000, Affinity, AF6471), Bax (1:1000, Affinity, AF0120), Bcl-2 (1:1000, Affinity, AF6139), Drp1 (1:1000, Affinity, DF7037), MFF (1:1000, Affinity, DF12006), MAVS (1:1000, Affinity, AF8074), XIAP (1:1000, Affinity, AF6368), MID51 (1:1000, Affinity, DF12019), MID49 (1:1000, Affinity, DF12044), and GAPDH (1:5000, Affinity, AF7021). After washing with TBST, membranes were incubated with HRP-conjugated goat anti-rabbit secondary antibody (1:5000, Bioss, bs-0295G-HRP) for 1h at room temperature. Protein bands were visualized using ECL reagent (Millipore, WBKLS0500) and quantified using Image J software.

### 4.5 Confocal laser immunofluorescence staining

Cell or tissue samples were first permeabilized with 3% H_2_O_2_, washed with PBS, and blocked with blocking solution for 2h at room temperature. Subsequently, samples were incubated with 1:200 diluted MFF (Affinity DF12006) and MAVS (Affinity DF12211) primary antibodies overnight at 4°C in a humidified chamber. After temperature recovery for 30min and PBS washing the next day, 1:200 diluted fluorescent secondary antibodies were added and incubated for 2h at 37°C in the dark. After PBS washing, DAPI nuclear staining was performed for 10min, followed by mounting with anti-fluorescence quenching mounting medium. Images were acquired using a confocal laser scanning microscope (HC PL APO CS 40x/1.30 OIL) at 1024×1024 resolution. Three channels were used for imaging: channel one with 405nm laser (5% intensity) for DAPI nuclear staining (blue); channel two with 561nm laser (10% intensity) for MFF and VISA proteins (red).

### 4.6 ELISA analysis

Enzyme-linked Immunosorbent Assay (ELISA) was used to measure levels of interleukin-1β (IL-1β) (RX203076M, RUIXIN BIOTECH, China), interleukin-6 (IL-6) (RX203049M, RUIXIN BIOTECH, China), tumor necrosis factor-alpha (TNF-α) (RX202412M, RUIXIN BIOTECH, China), cardiac troponin I (cTnI) (RXJ202543M, RUIXIN BIOTECH, China), and creatine kinase-MB (CK-MB) (RXJ201007M, RUIXIN BIOTECH, China) in cardiac tissue homogenates and cell culture supernatants. According to the manufacturer’s instructions, the detection ranges were 7.5–240 pg/mL for IL-1β, 3.75–120 pg/mL for IL-6, 20–640 pg/mL for TNF-α, 7.5–240 pg/mL for cTnI, and 1.5625–50 ng/mL for CK-MB.

Briefly, 50 μL of standards or appropriately diluted samples were added to pre-coated 96-well plates, followed by 50 μL of biotinylated detection antibody (except in blank wells), and incubated at 37°C for 60 minutes. After washing, 50 μL of enzyme-conjugate was added and incubated at 37°C for 30 minutes. Plates were washed five times, and 100 μL of substrate solution was added and incubated at 37°C in the dark for 15 minutes. The reaction was stopped by adding 50 μL of stop solution, and the absorbance was measured at 450 nm using a microplate reader.

The concentrations of target proteins in tissue homogenates were normalized to total protein content (pg/mg protein), which was measured separately using the bicinchoninic acid (BCA) protein assay. All samples were analyzed in triplicate, and each experiment was independently repeated three times.

### 4.7 Dual-luciferase reporter assay

Based on the predicted miR-27b binding sites from miRBase database, wild-type (Wt) and mutant (Mut) sequences of Mff 3’UTR were cloned into pmirGLO vector (Promega, E1330). HL-1 cells were seeded in 24-well plates at 1×10^5^ cells/well. When cells reached 60-70% confluence, they were co-transfected with miR-27b mimics (50nM) or mimics NC, and Wt-Mff or Mut-Mff reporter plasmids (1μg/well) using Lipofectamine 3000 (Invitrogen, L3000015). After 48h, luciferase activities were measured using Dual-Luciferase Reporter Assay System (Promega, E1910). Each group was tested in triplicate and experiments were repeated three times. Results were expressed as the ratio of firefly to renilla luciferase activity.

### 4.8 Transmission electron microscopy

Fresh heart tissue or treated cells were immediately fixed with 2.5% glutaraldehyde (in 0.1M phosphate buffer, pH 7.4) at 4°C for 4h, followed by post-fixation with 1% osmium tetroxide for 2h. After gradient ethanol dehydration, samples were embedded in Epon 812 resin. Ultrathin sections (60-70nm) were prepared using Leica UC7 ultramicrotome, double-stained with uranyl acetate and lead citrate, and examined under transmission electron microscope (Hitachi HI7800).

### 4.9 Cell viability assay

Cell viability was assessed using CCK-8 kit (Solarbio, CA1210). Cells were seeded in 96-well plates at 8×10^3^ cells/well in 100μL medium. After 24h culture, cells were treated accordingly. Following treatment, medium was replaced with 90μL fresh medium and 10μL CCK-8 reagent, and incubated at 37°C for 1h. Absorbance was measured at 450nm using a microplate reader, with blank wells for zero adjustment. Six replicates were set for each group and experiments were repeated three times. Cell viability (%) was calculated as: (OD related-OD blank)/(OD control-OD blank)×100%.

### 4.10 Cell transfection

HL-1 cells were divided into groups: control, LPS (1μg/mL), LPS+miR-27b agomir (100nM), LPS+miR-27b antagomir (200nM), LPS+miR-27b agomir+Ad5-Mff (virus titer 1×10^9^ PFU/mL), and LPS+miR-27b antagomir+Ad5-Mff shRNA (virus titer 1×10^9^ PFU/mL). MiR-27b agomir, antagomir and their negative controls were purchased from Ribobio. Ad5-Mff and Ad5-Mff shRNA adenoviruses were constructed by Shanghai GeneChem. Cells were seeded in 6-well plates at 2×10^5^ cells/well 24h before transfection. Transfection was performed using Lipofectamine 3000 according to manufacturer’s instructions. Medium was changed to complete medium 6h post-transfection. After 48h, cells were treated with LPS for additional 24h before collection for subsequent experiments.

### 4.11 Hematoxylin and eosin staining

After echocardiography, mice were sacrificed and heart tissues were immediately fixed in 4% paraformaldehyde (pH 7.4) for 24h. Tissues were dehydrated using an automatic dehydrator: 50% ethanol for 30min, 70% ethanol for 30min, 80% ethanol for 30min, 95% ethanol I for 30min, 95% ethanol II for 30min, absolute ethanol I for 30min, absolute ethanol II for 30min, xylene I for 30min, xylene II for 30min. Following paraffin embedding, 4μm sections were prepared using Leica RM2235 microtome. Staining procedure: deparaffinization in xylene I for 10min, xylene II for 10min; rehydration through graded ethanol (100%, 95%, 80%, 70%, 3min each); distilled water wash; Harris hematoxylin (Sigma, HHS16) staining for 6min; tap water wash for 5min; differentiation in 1% acid alcohol for 10s; bluing in tap water for 10min; 0.5% eosin Y solution (Sigma, E4382) staining for 3min; quick rinse in distilled water; dehydration through graded ethanol (70%, 80%, 95%, 100%, 1min each); clearing in xylene I for 5min, xylene II for 5min; mounting with neutral balsam (Sigma, G8410). Sections were examined and photographed under Olympus BX53 microscope at 400× magnification, with 5 random fields selected per sample for analysis. All imaging parameters were kept consistent, and morphological analysis was performed using Image J software.

### 4.12 Cell culture and model establishment

HL-1 mouse cardiac muscle cells were obtained from iCell Bioscience Inc. (Shanghai) and cultured in MEM (Gibco, 11095080) supplemented with 10% fetal bovine serum (FBS, Gibco, 10099141) and 1% penicillin/streptomycin (Gibco, 15140122). Cells were maintained at 37°C in a humidified incubator with 5% CO_2_and 70-80% relative humidity. Complete medium was changed every 2–3 days, and cells were passaged at 80-90% confluence using 0.25% trypsin-EDTA (Gibco, 25200056) for 2–3 minutes with a split ratio of 1:3. For SCM model establishment, HL-1 cells at 80% confluence were treated with LPS (from E. coli O111:B4, Sigma, L4391, 1μg/mL) for 24h (Zhang et al., 2022). Prior to treatment, cells were synchronized in serum-free medium for 12h, and treatment was conducted in low-serum medium containing 1% FBS. Each experiment was performed with 6 replicates and repeated independently three times.

### 4.13 Bioinformatics analysis

The GSE79962 dataset, containing transcriptomic analysis of cardiac tissues from patients who died of sepsis (n=20) and non-failing human donor hearts that could not be transplanted for technical reasons (n=11), was downloaded using the GEOquery package in R language. Differentially expressed genes were analyzed using the Limma package.

### 4.14 Statistical analysis

Data were analyzed using GraphPad Prism 9.0 software. Results are presented as mean ± standard deviation (s.d.). Comparisons between two groups were performed using two-tailed Student’s t-test. Multiple group comparisons were conducted using one-way analysis of variance (ANOVA) followed by Tukey’s *post hoc* test. *P*<0.05 was considered statistically significant.

### 4.15 Sample source utilization in this study

Samples from different sources were used for specific research purposes, ensuring multi-level and comprehensive experimental evidence. Clinical serum samples (from SCM patients, sepsis-only patients, and healthy controls) were primarily used to validate the clinical relevance of miR-27b expression levels, detecting differences in circulating miR-27b levels through RT-qPCR technology. Mouse cardiac tissue samples were mainly used for the following aspects: (1) pathological morphological observation through H&E staining to verify the successful establishment of the SCM model; (2) detection of miR-27b, Mff, MAVS, and related inflammatory factors expression levels through RT-qPCR; (3) observation of ultrastructural changes in cardiomyocyte mitochondria using transmission electron microscopy. HL-1 cardiomyocytes were primarily used for *in vitro* molecular mechanism exploration and intervention experiments, specifically including: (1) establishment of LPS-induced SCM cell model; (2) intervention experiments with miR-27b mimics/antagonists and Mff overexpression/knockdown to verify the direct targeting relationship between miR-27b and Mff; (3) dual-luciferase reporter gene assay to confirm the binding of miR-27b to the Mff 3’UTR; (4) detection of changes in expression of inflammatory factors and apoptosis-related proteins through Western blot and immunofluorescence; (5) detection of cell viability changes through CCK-8.

## Data Availability

The datasets presented in this study can be found in online repositories. The names of the repository/repositories and accession number(s) can be found below: (http://www.ncbi.nlm.nih.gov/geo) (GSE79962).
