# Supplementary figures and images for: MicroRNA-27b alleviates septic cardiomyopathy by targeting the Mff/MAVS axis
Source: Front Cell Infect Microbiol. 2025 Jul 22;15:1588461. doi: 10.3389/fcimb.2025.1588461 (PMC12321784; doi:10.3389/fcimb.2025.1588461)

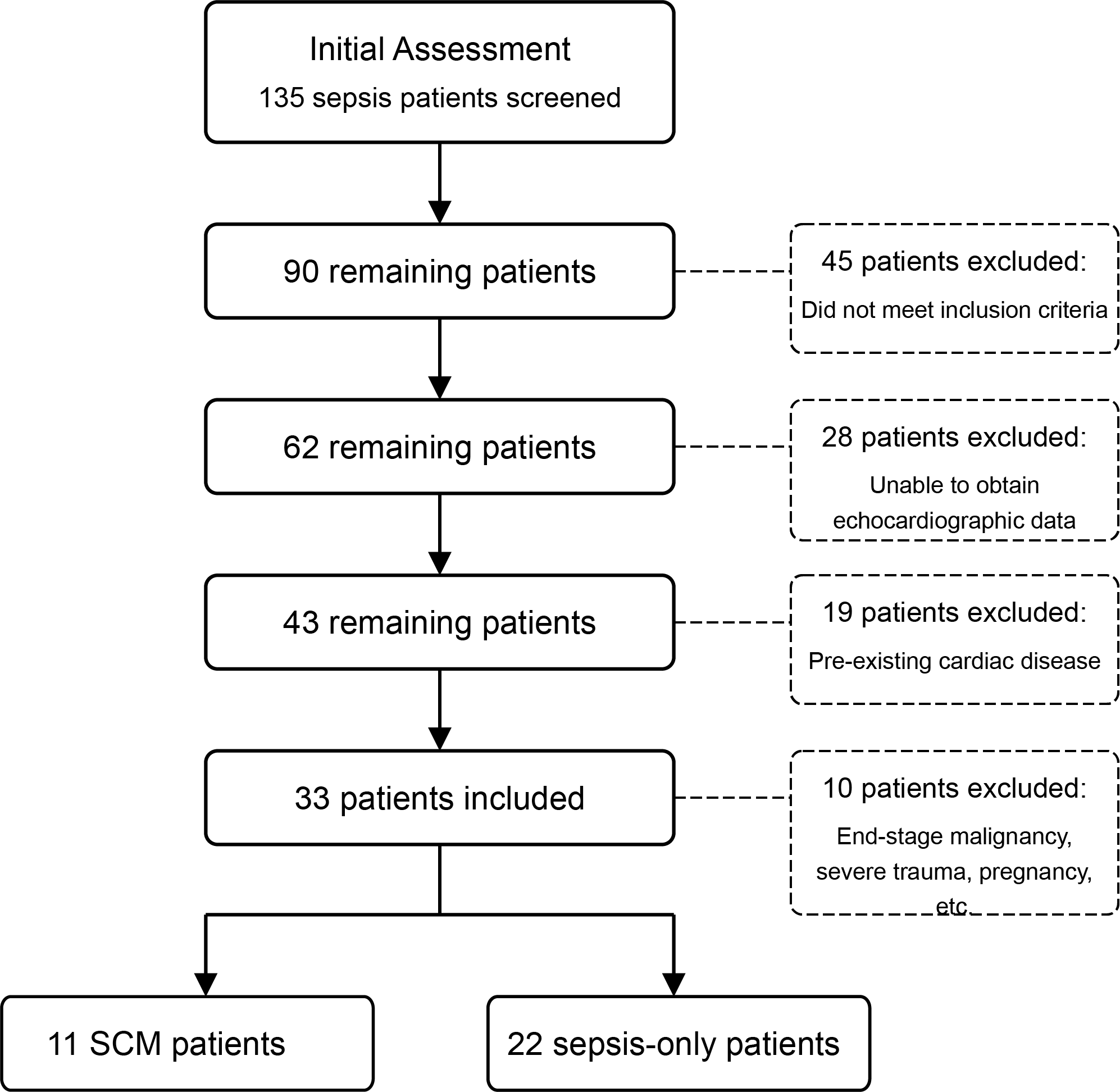

Supplement: Supplementary file 1 [file Image1.tif]
