# Supplementary material for: MicroRNA-27b alleviates septic cardiomyopathy by targeting the Mff/MAVS axis
Source: Front Cell Infect Microbiol. 2025 Jul 22;15:1588461. doi: 10.3389/fcimb.2025.1588461 (PMC12321784; doi:10.3389/fcimb.2025.1588461)
Supplement: Supplementary file 2 [file Table1.docx]

| **Characteristics** | **Sepsis (n=22)** | **SCM (n=11)** | ***P* value** |
| --- | --- | --- | --- |
| **Demographics** |  |  |  |
| Age, years* | 65.4 ± 2.5 | 69.5 ± 2.0 | ***0.001***† |
| Male sex, n (%) | 14 (63.6) | 7 (63.6) | 1.000§ |
| **Comorbidities, n (%)** |  |  |  |
| Diabetes | 8 (36.4) | 5 (45.5) | 0.722§ |
| Hypertension | 11 (50.0) | 6 (54.5) | 1.000§ |
| COPD | 2 (9.1) | 2 (18.2) | 0.584§ |
| CKD | 2 (9.1) | 2 (18.2) | 0.584§ |
| **Cardiac Parameters** |  |  |  |
| LVID, cm‡ | 4.9 (4.8-4.9) | 5.1 (5.0-5.2) | ***0.001***¶ |
| TAPSE, cm* | 2.0 ± 0.1 | 1.5 ± 0.1 | ***0.001***† |
| LVEF-S, %* | 59.8 ± 2.2 | 46.0 ± 1.6 | ***0.001***† |
| **Laboratory Values** |  |  |  |
| NT-proBNP, pg/mL* | 668.2 ± 90.3 | 913.2 ± 118.5 | ***0.001***† |
| cTnI, ng/mL‡ | 0.1 (0.1-0.2) | 0.6 (0.5-0.6) | ***0.001***¶ |
| Lactate, mmol/L‡ | 2.7 (2.5-3.0) | 4.2 (3.9-4.4) | ***0.001***¶ |
| PCT, ng/mL‡ | 5.5 (4.8-6.2) | 8.0 (6.9-8.8) | ***0.001***¶ |
| **Disease Severity** |  |  |  |
| APACHE II score‡ | 13.0 (11.0-15.0) | 22.0 (20.0-24.0) | ***0.001***¶ |
| SOFA score* | 5.5 ± 1.3 | 7.7 ± 0.8 | ***0.001***† |
| **Clinical Management** |  |  |  |
| Mechanical ventilation, n (%) | 8 (36.4) | 7 (63.6) | 0.163 |
| Vasopressor requirement, n (%) | 12 (54.5) | 8 (72.7) | 0.465§ |
| ICU length of stay, days‡ | 7.0 (6.0-8.0) | 14.0 (12.0-16.0) | ***0.001***¶ |

# **Table S1**. Baseline characteristics of study participants

*Data presented as mean ± standard deviation for normally distributed variables.
‡Data presented as median (interquartile range) for non-normally distributed variables.
†Independent t-test was used.
¶Mann-Whitney U test was used.
§Fisher's exact test was used.

**Abbreviations**: APACHE II, Acute Physiology and Chronic Health Evaluation II; CK, creatine kinase; CKD, chronic kidney disease; COPD, chronic obstructive pulmonary disease; cTnI, cardiac troponin I; ICU, intensive care unit; LVEF-S, left ventricular ejection fraction measured by Simpson's method; LVID, left ventricular internal diameter; NT-proBNP, N-terminal pro-brain natriuretic peptide; PCT, procalcitonin; SCM, septic cardiomyopathy; SOFA, Sequential Organ Failure Assessment; TAPSE, tricuspid annular plane systolic excursion.
